# The Relationship between Preparation and Biological Activities of Animal-Derived Polysaccharides: A Comprehensive Review

**DOI:** 10.3390/foods13010173

**Published:** 2024-01-04

**Authors:** Bochun Yang, Conghao Yang, Rui Liu, Wenjie Sui, Qiaomei Zhu, Yan Jin, Tao Wu, Min Zhang

**Affiliations:** State Key Laboratory of Food Nutrition and Safety, Food Biotechnology Engineering Research Center of Ministry of Education, College of Food Science and Engineering, Tianjin University of Science & Technology, Tianjin 300457, China; bcyang0810@126.com (B.Y.); yangconghao@mail.tust.edu.cn (C.Y.); lr@tust.edu.cn (R.L.); wjsui@tust.edu.cn (W.S.); jinyan@tust.edu.cn (Y.J.); zm0102@tust.edu.cn (M.Z.)

**Keywords:** animal polysaccharides, glycosaminoglycan, extraction method, yield, purification, structure, bioactivity

## Abstract

Polysaccharides are biomolecules found in microorganisms, plants, and animals that constitute living organisms. Glycosaminoglycans, unique acidic polysaccharides in animal connective tissue, are often combined with proteins in the form of covalent bonds due to their potent biological activity, low toxicity, and minimal side effects, which have the potential to be utilized as nutrition healthcare and dietary supplements. Existing studies have demonstrated that the bioactivity of polysaccharides is closely dependent on their structure and chain conformation. The characteristic functional groups and primary structure directly determine the strength of activity. However, the relationship between structure and function is still unclear, and the target and mechanism of action are not fully understood, resulting in limited clinical applications. As a result, the clinical applications of these polysaccharides are currently limited. This review provides a comprehensive summary of the extraction methods, structures, and biological activities of animal-derived polysaccharides that have been discovered so far. The aim is to promote developments in animal active polysaccharide science and provide theoretical support for exploring other unknown natural products.

## 1. Introduction

**Figure 1:**
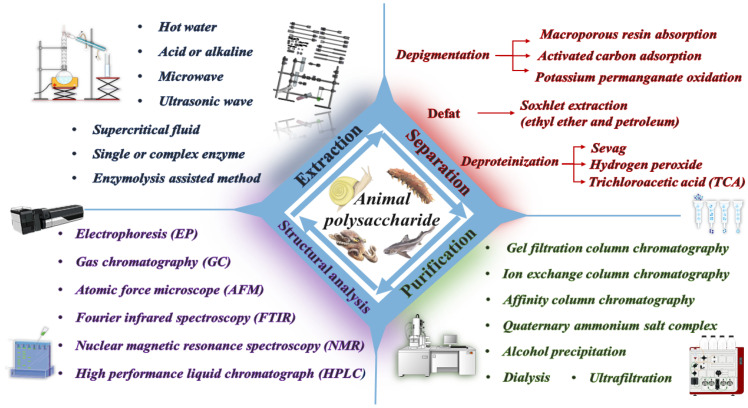
Main research processes of animal polysaccharides.

Polysaccharides are carbohydrates formed via the dehydration and condensation of multiple monosaccharides linked together by glycosidic bonds [1]. The molecular weights can reach tens of thousands or even millions, and they are considered one of the fundamental substances that constitute life activities and maintain biological functions besides proteins and nucleic acids [2]. They exhibit diverse branching compositions, molecular weights, and conformations [3]. Glycosaminoglycans (GAGs), also known as mucopolysaccharides, are linear polyanionic compounds that belong to the family of animal polysaccharides, which are polymerized from hexuronic acid (except keratan sulfate) and hexosamine repeating units [4]. Proteoglycans (PGs) are glycosylated proteins whose structure consists of one or more GAGs covalently linked to a core protein [5]. Serving as indispensable constituents of PGs, GAGs are regally divided into five kinds: hyaluronic acid (HA), chondroitin sulfate (CS), dermatan sulfate (DS), keratan sulfate (KS), and heparan sulfate (HS) along with heparin (Hep) [6]. Cell growth and development, intercellular homeostasis, and repair and regeneration are all linked to GAGs in the extracellular matrix [7]. GAGs are predominantly sourced from aquatic organisms, including marine fish, shellfish, and mollusks [8]. These creatures live in hydrated buffer systems with small temperature differences and rich ionic species, and the polysaccharides synthesized in vivo have distinctive structural groups and physicochemical properties [9], which are found in a variety of aspects, such as drug carriers, cosmetics, and healthcare foods. Relationships between the conformation of polysaccharides and their activities have been reported. To elucidate these relationships, appropriate preparation and analytical techniques are required. A single method is not sufficient to characterize the structure of GAGs. Fourier-transform infrared (FTIR), nuclear magnetic resonance (NMR), high-performance liquid chromatography (HPLC), and other methods are usually used to orthogonally integrate the obtained data. The objective is to conduct a more in-depth analysis of animal polysaccharides. In this review, we summarize the methods of extraction, isolation, purification, structural characteristics, and biological activities of animal-derived polysaccharides (Figure 1).

## 2. Extraction Methods of Animal Polysaccharides

Animal polysaccharide extraction methods mainly include water extraction, acid–alkaline extraction, enzyme extraction, ultrasonic extraction, and combined extraction. Extraction serves as a vital step towards obtaining crude polysaccharides. The glycosidic linkage connection, monosaccharide type, and disaccharide composition of polysaccharides can be influenced by factors such as pH, temperature, and solvent used, which may interfere with subsequent structural analysis.

### 2.1. Water Extraction Method

Traditional water extraction is the most commonly used physical extraction method, which has the advantages of simple operation, ease of control, and low cost. The principle is that polysaccharides are extracted by submerging raw materials in boiling water for a period of time. This method utilizes the solubility of high-temperature water to accelerate the release of polysaccharides. According to polysaccharides, which are insoluble in organic solvents, ethanol-graded precipitation obtains crude polysaccharides. However, water molecules cannot fully destroy the cell membrane, resulting in the incomplete extraction and loss of polysaccharides and a low yield [10].

Zheng et al. compared four methods for extracting polysaccharides from lumpus cartilage using a fixed material–liquid ratio of 1:40. They found that the water extraction method heated at 80 °C for 4 h had the lowest extraction rate, at approximately 3.52% [11]. Luan extracted the crude polysaccharide of *Solenidae* using a boiling water bath. The material–liquid ratio was 1:30 for 2 h, and then the residue was boiled again with a material–liquid ratio of 1:20 for 1 h. The extraction rate of crude polysaccharide was 1.7% lower than that of the alkaline extraction method, but it maintained a higher total sugar content at 55.4% [12]. That is because the solvent was only water, and water-soluble polysaccharides were extracted through intermolecular forces to retain the original structure of polysaccharides as much as possible. Ticar et al. used 80 °C hot water to extract heads of silver-banded whiting twice, each time lasting for 1 h. However, the yield of GAGs only reached 0.8% [13]. Getachew et al. modified the hot water extraction method using subcritical water (SWE) at approximately 125 °C to extract polysaccharides from Pacific oysters for 15 min. The process was optimized, resulting in a significantly higher yield of approximately 18.42% [14]. Mohammadi et al. investigated the efficiency of extracting polysaccharides from abalone in a high-pressure autoclave extractor with subcritical water at different temperatures, and the polysaccharide content was found to be high in the range of 250–280 °C, with a peak at 250 °C [15]. We can observe that adjusting the water to a subcritical state, even for a short period, significantly increases the yield of polysaccharides. However, this method is more expensive and difficult to industrialize.

### 2.2. Acid and Alkaline Extraction Method

Compared to water extraction, alkaline extraction more effectively breaks down glycopeptide chains between PGs and forms salts with acidic polysaccharides so that water-soluble polysaccharides can be easily released. Chen et al. discovered that the yield of polysaccharides from *Andrias davidianus* skin mucus increased by 2.45% when extracted using a 0.4 mol/L NaOH solution for 2 h at 45 °C with a material–liquid ratio of 1:25, compared to extraction using a 0.3 mol/L HCL solution under the same conditions [16]. On the other hand, Lu et al. obtained the opposite conclusion that by increasing the temperature to 50 °C and extending the extraction time to 3 h, the yield of polysaccharides in the coelomic fluid of *Phasolosma esculenta* extracted using NaOH with a mass concentration of 1.5% was only 0.92% [17].

The alkaline extraction method can enhance the yield of polysaccharides within a certain concentration range. However, the disadvantage is that some aminopolysaccharides may be degraded by strong alkaline, resulting in the Walden inversion and desulfurization [18], affecting polysaccharides’ structure analysis and activity verification. Therefore, low-concentration alkaline can be used for extraction.

Similar to the alkaline extraction method, the use of the acid extraction method can help break the bonds between polysaccharides and bound proteins, thereby improving the yield and purity of polysaccharides. Cheng et al. first used water to extract polysaccharides from *Mytilus edulis.* They then extracted the residual precipitate in a 0.2 mol/L HCL solution at 60 °C for 30 min and added 10% Na_2_CO_3_ for a further alkaline extraction of the residues. The acid extraction yield was the lowest at 3.5%, and the antioxidant capacity of the extracted polysaccharides was also the weakest [19]. It can be concluded that high concentrations of acid or base can cleave glycosidic bonds, irreversibly affecting the spatial structure of polysaccharides and reducing their biological activity. Therefore, neither laboratory nor industrial preparations are used very rarely.

### 2.3. Enzyme Extraction Method

Under mild conditions of enzyme action, each enzyme binds specifically to its substrate, allowing the reaction to be specific, which can selectively hydrolyze or degrade the cell membranes, reducing intermolecular resistance and allowing polysaccharides to be effectively released [20]. Enzymes function optimally at a specific pH and temperature, breaking down polysaccharides into small molecule fragments, thus improving the yield. Enzymes utilized in the preparation include alkaline protease, papain, neutral protease, etc.

Papain is the most commonly used enzyme for the extraction of animal polysaccharides. Maccari et al. selected papain for CS extraction from four species of bony fish by stirring overnight at 60 °C in a Na–acetate buffer solution at pH 5.5, and the highest yield among the four CS was only 0.34% [21], which is contrary to the findings of Zhang’s study [20]. The reason for this discrepancy may be because no screening of the optimum proteases was carried out prior to the extraction of polysaccharides. Bai et al. employed a trypsin–papain in a 0.1 mol/L Tris-HCL buffer at 55 °C to co-enzymatically extract the polysaccharides from the Lapemis curtus skin for 3 h with a crude extraction rate of 200 mg/g [22]. Wang also used both enzymes to extract GAGs from the Acipenser schrenckii cartilage, with a yield of 25% [23]. Wang et al. extracted CS from six marine animals using trypsin digestion at 37 °C for 4 h, followed by papain digestion at 60 °C for 4 h. The yield of CS was only 16.36% for *Raja porosa*, while the yield from the other five animals was less than 1%. This difference in yield may be due to the limitation that only *Raja porosa* is suitable for extraction using this method, and more suitable conditions need to be explored for the extraction of other animal polysaccharides [24]. Yuan et al. compared the extraction rate of *Sinonovacula constricta* polysaccharide (SCP) using a neutral protease extracted at 50 °C for 173 min and water extraction at 80 °C for 4 h. The enzyme extraction method resulted in a 12.26% higher yield than the water extraction method, but there was no significant difference in composition [25]. These findings suggest that enzyme extraction is a green and efficient method that can improve extraction rates while ensuring the integrity of the polysaccharide structure. Guo et al. extracted abalone viscera polysaccharide using an alkaline protease for enzymolysis at pH 9.5 for 19 h. They then selected a flavor enzyme at pH 7.0 from five enzymes for secondary enzymolysis. The extract had a high polysaccharide content of 51.75% and significantly reduced protein content [26]. The reason for this is the high efficiency of protein hydrolysis via alkaline protease and the fact that different binding sites of the flavor enzyme and alkaline enzyme facilitate further hydrolysis of polysaccharides and the removal of protein complexes.

Single enzyme extraction, complex enzyme extraction, and multiple enzyme segmented extraction methods are currently the most suitable for extracting animal polysaccharides.

### 2.4. Combined Extraction Method

In addition to the previously mentioned methods, several sustainable technologies have been steadily implemented, including microwave extraction, ultrasonic extraction, and supercritical fluid extraction. Each method possesses unique characteristics, depending on its specific mechanisms. Various methods were employed to obtain the most efficient extraction of animal polysaccharides, where enzymolysis, in conjunction with other methods, was mainly utilized. This method shortened the extraction time and increased the yield simultaneously.

Wang et al. compared the effects of enzyme, ultrasound, and ultrasound-assisted enzyme methods on the yield of polysaccharides from sea cucumber gonads. The results showed that the polysaccharide extracted via pH 7.5, 50 °C enzymolysis for 4 h followed by 400 Watt ultrasonic for 50 min had a higher sulfate acid group content, and the yield was better than the other two methods, about 6.09% [27]. Guo et al. conducted a study on the extraction of polysaccharides from various parts of *Scophthalmus maximus* using two methods: first, alkaline followed by enzyme, and second, enzyme followed by alkaline [28]. The results show that enzymes are used to hydrolyze the peptide bond first, which degrades the protein into small peptides and thus separates it from other substances. After that, the β-elimination reaction occurred under alkaline conditions, and the -O-glycopeptide chain was cleaved, which further released the polysaccharide from PG. Chen et al. optimized the specific parameters of the ultrasound-assisted enzymatic extraction of polysaccharides from thick-shelled mussels: acid protease was added to the solution, the extraction was performed at 64 °C for 36 min, and the ultrasonic power was 60 w [29]. The molecular weight of the polysaccharides obtained using this method was 1/10 of that obtained using hot water extraction, suggesting that ultrasonic treatment led to the degradation of polysaccharides. Although there is a decrease in molecular weight, no difference in monosaccharide composition was observed. Li et al. used a dual-phase saline extraction method papain, enzymatic extraction of polysaccharides from abalone guts while removing pigments and some heavy metal ions to shorten the time of purification and simplify experimental steps. The optimal extraction conditions were 30% ethanol and 12% Na_2_CO_3_. Under this condition, the yield of abalone visceral polysaccharides was 3.76% [30].

The utilization of multiple methods creates new possibilities for the extraction of polysaccharides. Crude extraction rates and purified molecular weights of polysaccharides from animal-derived sources are summarized in Table 1.

## 3. Method of Isolation and Purification of Animal Polysaccharides

The content of polysaccharides in animals is low and does not exist alone. Crude extracts contain numerous lipids, proteins, and inorganic salts, thus making pretreatment, including separating and removing impurities, an essential step in the polysaccharide purification process [55].

Pigmentation affects the chromatographic analysis of polysaccharides, thus affecting the accurate identification of chemical bonds. For the decolorization of pigmented polysaccharides, activated carbon or macroporous resin adsorption is commonly used [56]. Soxhlet extraction is employed using organic solvents such as diethyl ether and petroleum ether to degrease polysaccharides. The above two substances need to be carried out before extracting crude polysaccharides to avoid non-polysaccharide substances interfering with separation and purification.

Proteins are the most common impurities in aqueous environments because they covalently bind to polysaccharides dissolved in water, forming stable complexes. Sevag, TCA, and H_2_O_2_ are mostly used for protein denaturation, and a large number of hydrophobic groups are exposed and aggregated, which become insoluble precipitates and are removed [57]. Meanwhile, it should be noted that the Sevag method requires a repeated operation, resulting in a significant loss of polysaccharides and the residue of toxic chemical reagents; the other two methods react violently and easily cause polysaccharide degradation. Xin et al. used single-factor experiments to screen out the most suitable deproteinization conditions for crude polysaccharides from *Tenebrio Molitor*: deproteinization twice, each time with continuous shaking for 17 min and standing for 19 min, where the protein removal efficiency reached 61.25%. The influence degree of removal rate was determined via response surface optimization: deproteinization time > shaking time > standing time [58].

For polysaccharides that do not mix with organic solvents, we employed the alcohol fractional precipitation and quaternary amine salt complexation method to increase the concentration of polysaccharides [59]. The solubility of the same kinds of polysaccharides obtained via alcohol precipitation is similar, and the composition of monosaccharides may be different. For polysaccharide solutions containing low molecular weight organic matter or inorganic salts, select dialysis bags with suitable molecular weight for dialysis to remove contaminants. The principle of ultrafiltration is akin to that of dialysis, whereby membranes of various sources and pore sizes intercept substances with specific molecular weights, realizing the separation of large and small molecules. The properties of the solution before and after ultrafiltration do not change, with a low energy consumption and a high recovery rate. Li et al. conducted a graded alcohol precipitation of polysaccharide extract from Manila clam *Ruditapes philippinarum*. The highest polysaccharide extraction rate of 4.23% and the highest purity of 73.46% were achieved when the ethanol concentration was increased from 20% to 60%. However, when the ethanol concentration increased to 80%, the extraction rate and recovery decreased. [60].

High-purity polysaccharides are fundamental for elucidating structure–function correlations. Column chromatography was selected to purify samples. This technique takes into account the physicochemical properties of polysaccharides and separates the target products step by step by employing the mechanism of quantitative separation between stationary and mobile phases. There are three kinds of packing material: gel-filtration column chromatography, ion-exchange column chromatography, and affinity column chromatography. Ion-exchange chromatography (e.g., DEAE-52 cellulose) separates neutral and acidic polysaccharides based on ionic charge. Gel chromatography (e.g., Sephdex G-100), also known as molecular-exclusion chromatography, is based on the principle of using the molecular sieve effect for the elution of fractions with different molecular weight sizes. After the crude polysaccharide solution is eluted via polarity using ion-exchange chromatography, a single fraction is collected for secondary purification via gel chromatography to enhance the purity of polysaccharides. Animal-derived GAGs are acidic polysaccharides that can be gradually extracted by elevating ion concentrations (salt concentrations).

Polysaccharides isolated and purified using different methods vary considerably in properties and functions. Priority should be given to ensure that selected methods do not damage the main structures of polysaccharides, and then high-feasibility methods should be selected to carry out experiments. Conventional methods result in serious environmental pollution, so it is imperative to develop new “green” technologies to achieve the purification of polysaccharides in the future.

## 4. Structural Characterization of Animal Polysaccharides

The research and development of polysaccharides in terms of function, therapeutic properties, and toxicities are synchronized and continuous [61]. Without mastering the structure of isolated and purified polysaccharides, even if they have strong biological activity and safety, it is impossible to carry out pharmacological and toxicological studies, not to mention synthetic or structural modification, which hinders the development of new, high-quality drugs. Polysaccharides exhibit distinctive chain conformations, with structures ranging from spherical to helical, coiled, and rodlike shapes. Moreover, they contain (1→3), (1→2), (1→4), and (1→6) linkages and diverse α and β conformations [1]. The primary structures serve as the foundation for advanced structures. Currently, the identification of polysaccharide structures involves the relative molecular mass, the monosaccharide composition, the ratio of the number of substances, sugar ring conformations, anomeric carbon configurations, linkages between sugar residues, etc. Primary structures of polysaccharides are often analyzed using high-performance liquid chromatography (HPLC) [62], gas chromatography (GC), gas chromatography–mass spectrometry (GC-MS), Fourier-transform infrared spectroscopy (FTIR) [63], an atomic force microscope (AFM) [64], nuclear magnetic resonance spectroscopy (NMR) [65], and other methodologies.

### 4.1. Monosaccharide Composition

Oligosaccharides are highly water-soluble and more likely to enter the organism through multilayer membrane barriers to carry out their function. Acid hydrolysis, acetolysis, methanolysis, and other methods can degrade polysaccharides and explain their primary structure and active characteristics at a low molecular level [66]. HPLC is most suitable for glycan identification, especially for detecting the molecular weights of several thermosensitive glycans and polysaccharides. This technology is highly sensitive, requires only a small sample, has excellent reproducibility, can qualify components based on the retention time of spectra, and can quantify the peak area.

Wang isolated polysaccharides from various sea gonads and discovered that the monosaccharides consisted mainly of mannose (Man), glucosamine (GlcN), and glucose (Glc), with small amounts of rhamnose (Rha), glucuronic acid (GlcA), galactosamine (GalN), galactose (Gal), and fucose (Fuc) together [51]. Bai conducted a compositional analysis of CS in the sturgeon bone. The analysis revealed that the major constituents were Glu and GalN, with a proportion of 1.04:1 comprising 48.23% and 46.29% of the sample, respectively. A peak different from the monosaccharide standard was also captured, presumably a disaccharide produced via antacidolysis [35]. Yim et al. analyzed the monosaccharide composition of polysaccharides extracted from abalone and observed that the crude product had a higher fucose than other monosaccharides, which may be responsible for its antiviral activity [67]. Wang et al. summarized and generalized available bioactive polysaccharides and found that sulfate groups and fucose were related to anti-inflammatory activity; Man was related to immunoregulatory activity, and rhamnose mainly affected antioxidant and antimicrobial activities of polysaccharides [68], which were specific to the structure of animal polysaccharides (Table 2).

### 4.2. Functional Groups and Chemical Bonds

After determining the compositions and molecular weights of the monosaccharides, FTIR was employed to detect the functional groups obtained in the polysaccharides. FTIR is applied for the qualitative analysis of unidentified substances, and spectra are based on the vibration or rotational excursion of atoms comprising chemical bonds or functional groups. According to the origin of the absorption peaks, they can be divided into the functional group region (2.5–7.7 μm, i.e., 4000–1330 cm^−1^) and the fingerprint region (7.7–16.7 μm, i.e., 1330–400 cm^−1^). The absorption peaks within the functional region arise from stretching vibrations of groups and are few, which are used to identify functional groups. The situation is different in the fingerprint region, where the peaks are numerous and complex, not typical, and are mainly generated via the stretching vibrations of some single bonds C-O, C-N, and C-X (halogen atoms) and bending vibrations of hydrogen-containing groups like C-H and O-H, as well as via vibrations of the C-C skeleton. When there are slight variations in the molecular structures, absorption peaks in this area will vary.

Gao et al. discovered that the purified polysaccharides from snail mucilage were pyranose, exhibiting distinct absorption peaks between 810 and 780 cm^−1^. The absorption peak at 1426 cm^−1^ is due to the stretching vibration of the C-O bond on the residue of iduronic acid, confirming the presence of -COO-. Furthermore, the presence of -COO- and CH_3_CO- is evidenced by a strong characteristic absorption peak of the C=O bond at 1640 cm^−1^. Taken together, these groups indicate that the polysaccharide is a typical GAG [52]. Similarly, Wang et al. also identified the snail mucilage polysaccharide as a β-pyranose by finding a characteristic β-glycosidic bond absorption peak at 891 cm^−1^ [69]. Krichen et al. analyzed two purified fish skin sulfated polysaccharides, SHSP and GTSP, using IR, and the characteristic absorptions at 1382 cm^−1^ and 1394 cm^−1^ confirmed the presence of a C-O-S bond in the sulfate group. A pyranose unit is suggested via absorptions at 1152 and 1034 cm^−1^ (SHSP) and 1156 and 1106 cm^−1^ (GTSP) [70]. Song et al. conducted an analysis of the IR of polysaccharides from Patinopecten yessoensis viscera and attributed the broad peak at 3428.4 cm^−1^ to the stretching vibration of the -OH bond, while the multiple peaks at 1100–1000 cm^−1^ belong to the stretching vibration of the C=O and C-C bonds in the pyranoid ring, as well as the stretching vibration of the glycosidic bond C-O-C. The peak near 1247.6 cm^−1^ is caused by the stretching vibration of the S=O bond of the sulfate group and is typical of sulfate polysaccharides [46].

### 4.3. Disaccharide Composition

NMR is used as an analytical chemistry technology to study the molecular structures and conformation of polysaccharides. Its principle involves identifying groups based on their chemical shifts, determining linkage and sequence of residues through numbers of coupled splitting peaks and coupling constants [71], and analyzing proton ratios of each group through H-peak integral areas. The study of polysaccharides is generally centered around Proton nuclear magnetic resonance (^1^H NMR), Carbon-13 nuclear magnetic resonance (^13^C NMR), correlation spectroscopy (COSY) in a heteronuclear single quantum coherence (HSQC), as well as a heteronuclear multiple-bond correlation (HMBC).

Zuo analyzed the disaccharide composition of CS extracted from tilapia processing by-products. The findings indicate that CS-C are present in both fish heads and tails, with the CS in tails containing ∆Di-2S,6S, while disulfide disaccharides are absent from the CS in heads. The composition of CS in fish spines and fins was similar, but CS in spines does not contain ΔDi-2S,6S, and CS in fins does not contain ΔDi-4S,6S. Most of the absorption peaks of ^1^H NMR appeared in three regions. The first region showed a characteristic peak of CS, while the middle region mainly consisted of sugar ring proton signals. The anomeric proton signal at 5.3 ppm in the last region was assigned to the C=O bond, thus confirming the existence of N-acetylglucosamine [47]. Signals elsewhere in the region were consistent with the analysis of disaccharide.^13^C NMR makes up for the shortcomings of ^1^H NMR. It can directly determine the molecular skeleton, giving information on various carbon-containing functional groups. The oyster polysaccharides extracted using subcritical water by Getachew et al. were analyzed as follows: the absorption peak at 1150 cm^−1^ was due to α-(1→4) glycosidic bonding. The stretching of the C-O bond appeared at 1020.16 cm^−1^, while the 1077.04 cm^−1^ peak was attributable to the deformation vibration of the -OH group. The presence of a characteristic peak at 848.52 cm^−1^ indicated the presence of oyster polysaccharides in an α-configuration. In ^13^C NMR, there were no chemical shifts above 100 ppm, indicating the absence of acetyl and carboxyl groups. The absence of a furan ring C-1 resonance band within the 107–109 ppm range confirmed that the polysaccharide was pyranose. Additionally, the peaks detected in the 76.96–69.27 ppm and 60.50 ppm regions corresponded to an α-configuration of dextran’s C2-C5 and C6, respectively. Thus, based on the NMR spectrum, the polysaccharide sample could be confirmed to be in an α-configuration pyranose, corresponding to the FTIR analysis results [14]. Additionally, Seedevi et al. analyzed 2D NMR COSY spectra based on 1D NMR of GAG extracted from cuttlefish. The cross peaks between 4.50 and 4.75 ppm indicated the presence of unsubstituted amines H-1 and H-2 that contained N-acetylglucosamine residues within sulfate GAG, proving the presence of glucuronic acid. Moreover, the signals at 3.80, 3.87, and 3.29 ppm assigned to H-4, H-3, and H-2, respectively, identified the position of glucuronic acid residues in sulfate GAG [72].

### 4.4. Chain Conformation

SEM uses secondary electron signal imaging to observe the surface morphologies of samples, which can also be used for the qualitative and semi-quantitative analysis of components, with the advantages of a large imaging range and fast scanning speed. In contrast, the AFM avoids any special handling and preserves the physiological state and integrity of polysaccharide molecules as much as possible [27]. Its imaging technology reveals 3D information on molecular morphologies, sizes, and conformations of samples in a solution. Furthermore, it exploits the intermolecular force between atoms to emphasize sample properties, directly reflecting the sensitivity of conformation to the microenvironment.

Mou et al. extracted two sea cucumber polysaccharides, fCS-Hm and fCS-Aj, using an alkaline extraction–enzymolysis method. With a similar molecular weight but relatively low sulfate content, FCS-Hm demonstrated greater extended chain conformations than fCS-Aj. Since fCS contains complex sulfated fucose branches, in solution, it causes its chain to stretch outward, forming an extended linear chain conformation. fCS-Aj and fCS-Hm have a random coil and extended linear chain conformations, respectively [50]. Li et al. studied sea cucumber CS: fCS-Pg, fCS-Ib, fuc-Pg, and fuc-Ib from diverse origins, where Pg and Ib are two species, and fCS denotes fucosylated CS, whereas fuc stands for fucoidan sulfate. An analysis of AFM images revealed that all the CSs except for fuc-Ib exhibited random linear chains. Chain lengths ranged between 100 and 1000 nm with some spherical aggregates. Notably, fuc-Ib showed a completely spherical structure. The variation in CS morphologies could relate to its fucose branches [30]. Dong et al. visually observed CS from discarded codfish bones as a white flocculent with an irregular lamellar structure using SEM, and spherical particles were visible after magnification. An observation under an AFM was able to show that CS was an irregular spherical structure with uniform distribution. The height of the individual polysaccharides was generally between 0.1 and 1 nm, indicating that the samples were in an aggregated state [37]. Yang et al. observed the structure of purified SCVP-1 from sea cucumber viscera using SEM, which showed a loose and irregular lamellar structure when magnified 500 times using SEM and continued to magnify it up to 2000 times to observe a rough surface morphology, curling, and a few spherical aggregates. SCVP-1 was not completely uniform in size and aggregated to form surface protrusions, as observed using an AFM [38].

Most studies typically use a combination of SEM and AFM images to analyze the sample surface, using SEM for the initial exploration and an AFM for the higher-resolution images at the nm level to distinguish surface changes at the atomic level and calculate the roughness of the sample surface.

The aforementioned measures can complement each other and accurately define the real and clear structure of polysaccharides. Nevertheless, physicochemical properties and extraction procedures may impact morphologies, resulting in discrepancies in the polysaccharide analysis of the same species. A more rigorous system must be established in future research processes to standardize the extraction and purification of polysaccharides and to avoid any structural discrepancies caused by external factors.

## 5. Biological Activities of Animal Polysaccharides

**Figure 2:**
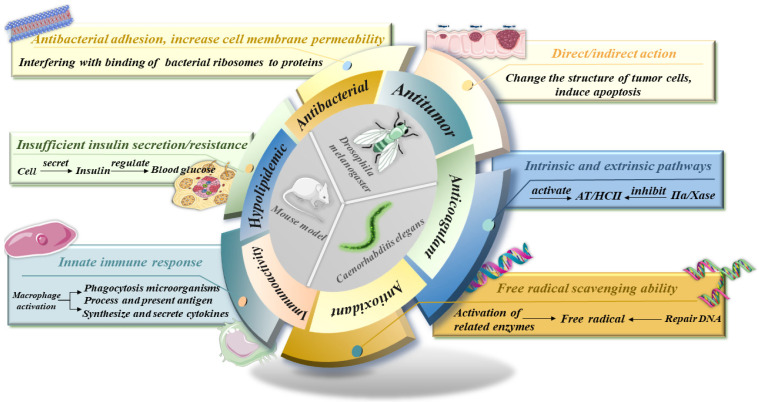
Main research processes of animal polysaccharides. (The central section of the circle shows animal models commonly used in experiments.).

In recent years, investigations on the bioactivities of animal polysaccharides have been emerging, and the following have been unearthed: hypoglycemic [73], hypolipidemic [74], anticoagulant [75], antiviral [76], and immunoregulatory activities (Figure 2). Within the life sciences field, we are committed to finding natural products with excellent biodegradability and compatibility, and polysaccharides are one of the representative substances [1]. An in-depth exploration of the active effects and mechanisms of action has facilitated the swift adoption of animal-derived polysaccharides in food science, biopharmaceuticals, industrial and agricultural production, and interdisciplinary fields. Based on this, we have listed the detailed data of some animal polysaccharide activity verification experiments to better compare the differences between polysaccharides and the same activity (Table 3).

### 5.1. Hypoglycemic Activity

The main etiology of primary diabetes is divided into two pathways: insufficient insulin secretion and the development of insulin resistance, which contribute to increased blood glucose levels and lead to the deterioration of the condition [81]. Current clinical drugs may induce other organ pathologies with long-term eating and have disadvantages of unremarkable efficacy or uncontrolled dosage. Similar to α-glucosidase inhibitors, polysaccharides can inhibit the expression of α-glucosidase, thereby reducing the body’s absorption of carbohydrates and achieving the purpose of lowering blood glucose levels. The hypoglycemic activity of animal polysaccharides brings hope for developing such drugs.

Elevated total cholesterol (TC), low-density lipoprotein cholesterol (LDL-C), TC/HDL-C, and decreased high-density lipoprotein cholesterol (HDL-C) are the most important factors in the development of coronary heart disease. Zhou et al. investigated the hypoglycemic activity of mudskipper polysaccharide (MAP) on streptozotocin (STZ)-induced mice. The hypoglycemic effect of high-dose MAP (200 mg/kg) on STZ-induced mice was comparable to that of the oral hypoglycemic agent metformin (150 mg/kg) by measuring fasting blood glucose. After 4 weeks of continuous administration, it showed results similar to the metformin-treated group, with lipids almost returning to normal levels. Type I diabetes in STZ-induced mice is caused by the destruction of pancreatic β-cells, reducing endogenous insulin secretion. It was demonstrated that appropriate concentrations (100 and 200 mg/kg) of MAP dose-dependently increased superoxide dismutase and glutathione peroxidase activities, which counteracted oxidative stress and inhibited the production of proinflammatory factors in mice [77].

The α-glucosidase in intestinal mucosa further degrades starch, and oligosaccharides, after decomposition into glucose, become monosaccharides, increasing blood glucose. Getachew et al. found that oyster polysaccharides inhibited the expression of α-glucosidase in a dose-dependent manner, significantly increased the half-maximal inhibitory concentration (IC_50_), and inhibited the carbohydrate-degrading enzyme (α-amylase) higher than α-glucosidase [13].

### 5.2. Hypolipidemic Activity

Excessive TG and TC in the blood are oxidized via free radicals to form lipid peroxides, which are deposited in the body and cause atherosclerosis. Secondary hyperlipidemia is a major cause of atherosclerosis. LDL-C is a transporter of cholesterol to peripheral tissues and has atherogenic capacity. HDL-C transports via the opposite pathway, and therefore, HDL-C has a protective effect against hyperlipidemia. Polysaccharides can bind to lipids and participate in cholesterol metabolism, acting as carriers to accelerate the transport of lipids in serum. Polysaccharides can also bind to LDL, promote the breakdown of TG, and accelerate cholesterol metabolism [78].

Wu et al. found that the hypolipidemic activity of fCS derived from different sea cucumbers may be related to the degree of sulfation of their fucose branches. Polysaccharides with 3,4-O-disulfation fucose branches were found to have more significant hypolipidemic activity and dose-dependently reduced the liver mass of mice compared to those with 2,4-O-disulfation fucose branches [78].

Li et al. investigated CS from different sea cucumbers: fCS-Pg, fCS-Ib, fuc-Pg, and fuc-Ib, the morphology of which was analyzed in detail in Section 4.4, and it can be summarized that only fuc-Ib exhibited a spherical structure, while the other three had a linear structure. Hyperlipidemia was induced in rats through high-fat diet (HFD) feeding, and it was discovered that administration of fCS and fuc was found to reduce both TG concentrations in the serum. In contrast, there was no significant reduction in TC in the serum before and after fuc-Ib intake. Fuc-Ib also had no effect on reversing HDL-C and LDL-C levels, which HFD disrupted. Conformational relationships of polysaccharides were speculated based on the experimental data: those containing sulfhydryl groups exhibit spherical conformations with reactive groups embedded at the chains’ center, which may weaken their contact with lipid regulation-related proteins and thus decrease their hypolipidemic activity. The other three polysaccharides with linear chain conformation have more active site contacts, alleviating the dyslipidemia induced via HFD to different degrees [82].

Studies have demonstrated a close correlation between obesity and dyslipidemia. Qi established a mouse model of HFD-induced obesity and orally gavaged different doses of CSs from sturgeon skulls and found that CS alleviated the elevation of fasting blood glucose indexes in mice at all doses within the range of determination and considerably reduced lipid accumulation in the HFD group. Glucose and insulin tolerance experiments demonstrated that CS improved HFD-induced impairment in mice. Elevated levels of TC and LDL-C due to HFD are key indicators of many cardiovascular diseases. Moreover, CS treatment reduces the expression of inflammatory factors and ameliorates the elevated levels of TC and LDL-C brought about by a high-fat diet, producing a positive anti-obesity effect [83].

### 5.3. Anti-Tumor Activity

China has undergone a rapid epidemiological transition in the past few decades. Regardless, cancer has consistently been a major contributor to the burden of disease in the country. Irregular dietary patterns have led to an increasing incidence of diseases, resulting in cancerous tumors and high mortality rates, with chemotherapeutic interventions being the common cancer treatments today. However, cancer metastasis following surgery and severe side effects caused by chemotherapy inflict significant suffering on patients. Polysaccharides can be combined with other drugs to inhibit tumorigenesis or metastasis and can also directly induce the apoptosis of cancer cells, which is very promising. Therefore, it is urgent to extract polysaccharides from animals with both anti-tumor activity and low toxicity [84].

Peng et al. investigated the impact of highly sulfated chondroitin sulfate E (CS-nE) in the cartilages of Dosidicus gigas on tumor metastasis. The study found that mice injected with 4T1 cancer cells that were treated with CS-nE displayed fewer tumor foci in the lungs and were lighter in weight, showing a dose-dependent inhibition in the low-dose range (50–100 μg/a mouse) compared to the control group [39].

In order to investigate the relationship between molecular weight and biological activity, Wu et al. prepared the sturgeon low molecular CS: SCS-f1, SCS-f2, and SCS-f3 using lyase AC. The experimental data demonstrated that the Caco-2 cellular uptake of enzymatically cleaved SCSs was significantly enhanced and showed a negative correlation with mass-average molar mass. In terms of anti-tumor activity, all SCS samples exhibited a dose-dependent inhibition of HT-29 colorectal cancer cell growth, but only SCS-f2 showed no significant toxicity to normal cells [79].

Li verified the inhibiting effect of GAG from visceral organs of *perna viridis* on K562 chronic myeloid leukemia cells, CNE-2Z human nasopharyngeal carcinoma cells, and the strongest inhibiting effect on S180 osteosarcoma cells, with inhibitory activity of 60.6% at 200 μg/mL, via in vitro culture [85].

### 5.4. Antioxidant Activity

Reactive oxygen species are by-products of normal oxygen metabolism. High concentrations of these species can cause an imbalance between oxidation and antioxidation in the body, leading to oxidative stress [86]. Oxidative stress is caused by a negative effect that disrupts the normal function of cellular lipids, proteins, and DNA, inducing diseases such as inflammation, cancer, neurodegenerative diseases, aging, and immune system damage. Certain animal-derived polysaccharides have been linked to antioxidant effects in today’s research through in vitro chemical and biological modeling [87].

Zhao and Qin examined the total antioxidant capacity (T-AOC) and free radicals scavenging capacity of the muscle, viscera, and body surface mucus derived from snails. The results showed that the highest T-AOC was present in snail mucus, and all three tissues exhibited significant DPPH-scavenging capacity [48]. Sun et al. found that the hydroxyl radical-scavenging ability gradually increased with increasing concentrations of GAG from *Mactra veneriformis*, and the scavenging ability increased significantly after 20 mg/mL before leveling off and reaching 100% scavenging at 30 mg/mL [44]. Chen et al. found that the polysaccharide fractions, purified from thick-shelled mussels, were effective in scavenging -OH, DPPH-, ABTS-, and -O2-. The scavenging ability increased with the rise in concentration and stabilized over time. However, the abilities of all scavengers were slightly lower than that of VC [29]. Zhu et al. discovered that purified abalone gonad polysaccharide demonstrated an improved ability to scavenge DPPH- and -OH radicals in a dose-dependent manner, which was superior to that of VC and VE, suggesting that this sulfated polysaccharide is a better free radical scavenger [36].

### 5.5. Anti-Aging Activity

Aging is a progressive and irreversible functional decline involving multiple organs and numerous responses that occur in all organisms [88]. GAG is primarily located in the extracellular matrix and is synthesized via fibroblasts within dermal cells. Its main function is to signal and maintain skin hydration and intercellular homeostasis. Skin aging is caused by a combination of internal factors, such as the body’s natural aging process and disease, and external factors, such as exposure to ultraviolet radiation, environmental conditions, and smoking [89]. Current research focuses on how to delay organismal or cellular senescence effectively, but drugs in clinical application still have shortcomings in long-term eating. *Caenorhabditis elegans* is primarily used as an animal model to validate the anti-aging activity of polysaccharides.

Gálvez-Martín et al. verified the anti-aging activity of HA from rooster combs. The group treated with HA significantly promoted the proliferation of primary human dermal fibroblasts and primary human epidermal keratinocyte cells. After 24 h of exposure, the 0.5 mg/mL HA-treated group increased by 14.81% compared with the control group. The cell viability of the 1 mg/mL HA-treated group exposed for 48 h was still increased by 2.08-fold compared with the control group, although it decreased. In addition, HA can effectively promote glycosaminoglycan (GAG) synthesis and prevent generating reactive oxygen-induced DNA damage [89]. Similarly, Alcântara et al. extracted HA from tilapia eyeballs, which was found to be non-toxic to cells and even stimulated the proliferation of fibroblasts [90]. Based on these experiments, it can be concluded that HA from animals has anti-aging properties.

Zuo extracted CS from various parts of tilapia, including heads, fins, spines, and tails, and confirmed its anti-aging properties. The results demonstrated that CS from the four parts showed a dose-dependent reduction in heat stress damage in *Caenorhabditis elegans*, indicating that the extracted CS could delay *Caenorhabditis elegans*’ death at a certain dose. Notably, fish heads demonstrated the most significant CS anti-aging activity, while the fins exhibited the weakest effects. Based on this finding, it was found that CS from the fish heads treated group prolonged *Caenorhabditis elegans*’ lifespan, reduced the accumulation of lipofuscin in *Caenorhabditis elegans*’ body in a dose-dependent manner, and maintained the health of the organism by regulating the expression of relevant aging genes [47].

### 5.6. Antibacterial Activity

The antibacterial mechanism of polysaccharides involves interfering with bacterial ribosomes binding to proteins, impeding their normal growth and metabolism, inhibiting adhesion, and enhancing permeability by disrupting cell membranes [91]. Natural plant polysaccharides exhibit antibacterial activity through artificial modifications, including sulfation and acetylation. Most aquatic animal polysaccharides contain sulfate groups, which can be experimentally verified whether they have antibacterial properties or not and accelerate the process of polysaccharide research.

Krichen et al. validated the antibacterial activity of sulfated polysaccharides extracted from the skin of gray triggerfish (GTSP) and smooth hound (SHSP) and purified individual fractions, respectively. For the crude product, antibacterial activity showed an upward trend with increasing polysaccharide concentration, with the most significant inhibitory effect on *Salmonella enterica* among Gram-negative bacteria and no inhibitory effect on *Enterococcus faecalis* among Gram-positive bacteria and *Escherichia coli* among Gram-negative bacteria. The inhibitory activity of SHSP was superior to that of GTSP against all the food-pathogenic bacteria. Regarding antifungal activity, all high-concentration treatment groups significantly inhibited *Fusarium solani* but showed no inhibition against *Botrytis cinerea*. Three single fractions were obtained for both polysaccharides, with no significant difference between the three fractions in GTSP, while the chemical structures of the three fractions in SHSP were completely different. However, both second fractions, F_GII_ and F_SII_, showed the most significant inhibitory effect on bacteria [70].

Jridi J et al. compared GAGs in cuttlefish skin and muscle, and antimicrobial activity was not the same due to different degrees of sulfation. While the total sugar and uronic acid content of muscle polysaccharides were higher, the skin polysaccharides exhibited better antibacterial activity. The minimum inhibitory concentration of *S. enterica* and *E. coli* was 0.78 and 1.56 mg/mL, respectively [92].

The high infectivity and pathogenicity of the SARS-CoV-2 virus have been identified. Yim et al. discovered that crude polysaccharide extracted from abalone demonstrated slight toxicity at a 1 mg/mL concentration. Moreover, the polysaccharide inhibited a cellular infection with the SARS-CoV-2 pseudovirus, COV-PS02, at different concentrations. This suggests polysaccharide has antiviral activity and may be isolated and purified as a COVID-resistant agent [67].

### 5.7. Immunological Activity

The immune function of an organism is achieved via interacting lymphocytes, monocytes, and other related cells and their products [93]. Macrophages initiate and participate in an innate immune response, and after activation, they can phagocytose pathogenic microorganisms [94], process and present antigens, and synthesize and secrete cytokines to enhance the body’s immune defense capability. Yang et al. found that sea cucumber viscera sulfated polysaccharides (SCVP-1) had a dose-dependent effect on macrophage proliferation, and at a concentration of 400 μg/mL, the cell viability rate was as high as 119.2%, approximately, and the cell growth rate was increased by nearly 20%. Because activated macrophages can secrete a series of chemokines in the inflammatory response, the effect of SCVP-1 on them continued to be verified. Data show that SCVP-1 induces chemokine secretion through an up-regulation expression of iNOS, IL-1β, IL-6, and TNF-α. The mRNA expression of rats treated in the high-dose group (400 μg/mL) was 8–10 times higher than in the control group [38].

Yu et al. discovered that both purified GAGs from the skin of *Rana dybowskii* Guenther in the high-dose treatment group (400 μg/mL) increased the expression of cytokine NO and TNF-α in macrophages in the peritoneal cavity of mice, thus enhancing macrophage phagocytosis [31]. Yin et al. reached a similar conclusion for GAGs (HGAG) extracted from *Apostichopus japonius*, which promoted the proliferation of mouse splenic lymphocytes and the production of serum hemolysin in a dose-dependent manner. Furthermore, treatment with different dosages of HGAG effectively promoted delayed-type hypersensitivity in mice [80].

Li demonstrated that *Perna viridis* GAG (PVG-1) at 100 μg/mL had the best enhancement of NO production via macrophages in the mouse peritoneal cavity. At the same dose, the lymphocyte stimulation index in mice of pure PVG-1a was higher than that of PVG-1, and the stimulation index of PVG-1a was the highest at 100 μg/mL. The results were similar regarding NK cell-killing ability, and the activity of PVG-1a at 50 μg/mL was the highest in the experiment [79].

### 5.8. Anticoagulant Activity

After tissue suffers a physical injury, activating factors at the injury site stimulate platelets to aggregate and become clots, which play a role in initial hemostasis. The anticoagulant activity, one of the most studied properties of GAGs, inhibits the conversion of prothrombin to thrombin and has an anticoagulant effect [95]. The imbalance between coagulation, anticoagulation, and abnormal coagulation–fibrinolysis can lead to thrombosis. Activated partial thromboplastin time (APTT) is a performance indicator of the effectiveness of the intrinsic coagulation pathway and is commonly used as a preliminary test for molecules with anticoagulant effects [96]. Thrombin time (TT) reflects the fibrinogen level in plasma and amounts of heparin-like molecules in plasma. It is used to detect heparin dosage, which is a common pathway of coagulation [97].

Chen et al. found that all four sea cucumbers with different origins had anticoagulant activity and were effective in prolonging APTT and TT even at low concentrations [40]. Peng et al. extracted high CS-nE from cartilages of *Dosidicus gigas*, which were found to have a bleeding capacity lower than that of heparin compared to the heparin standard [39]. Song et al. verified the anticoagulant activity of purified fractions SVP2-1 and SVP2-2, polysaccharides from *Patinopecten yessoensis* viscera. Using heparin as a control, both SVP2-1 and SVP2-2 were found to inhibit fibrin at all concentrations, impeding the conversion of fibrin via active thrombin and positively affecting blood flow. SVP2-2 demonstrated greater activity than SVP2-1, likely due to its higher sulfate group content. Moreover, at concentrations ≥ 1.0 mg/mL, SVP2-2 almost completely inhibited fibrin formation. Both SVP2-1 and SVP2-2 prolonged APTT and TT in a concentration-dependent manner at concentrations of at least 10 μg/mL [46]. Liu et al. prepared fCS from two sea cucumbers with different molecular weight fragments and investigated their differences. In vivo experiments revealed insignificant anticoagulant activity between the two fCS with different fucose branches. However, the difference in activity of polysaccharides with different molecular weight fragments originating from one sea cucumber was obvious. This study provides a new perspective on the conformational relationship of polysaccharides [98].

## 6. Conclusions

The widespread availability, hydrophilicity, and excellent biocompatibility of polysaccharides have rendered them the preferred material in the food, pharmaceutical, textile, and paper industries. Animal-derived polysaccharides are added as ingredients in cosmetics to serve for moisturizing and skin protection. Chitosan can be developed as an eco-friendly adsorbent for eliminating hazardous heavy metal ions from industrial wastewater. Using various combinations of polysaccharides to encapsulate beneficial microorganisms—a process known to heighten their survival rate—presents an auspicious method for biological control against plant pathogens. Moreover, chondroitin sulfate extracted from animal cartilage exhibits superior wound-healing abilities and finds extensive application in pharmaceuticals and nutraceuticals. 

Sulfate groups and fucose fractions can significantly impact the biological activity of animal polysaccharides, potentially altering their functionality. For instance, the utilization of sulfated polysaccharides with anticoagulant properties in the development of drugs can effectively prevent thrombosis and have positive therapeutic effects on patients with stroke and chronic obstructive pulmonary disease. Furthermore, sulfated polysaccharides can chelate metal ions and act as antioxidants by scavenging superoxide anions and free radicals. This attribute can be exploited in formulating cosmetics and healthcare products to cater to specific target populations. Additionally, sulfated polysaccharides possess excellent antiviral properties and can modulate the immune response by regulating the signaling pathway induced by macrophages. The consumption of drugs containing sulfated polysaccharides can significantly enhance human immunity, particularly in individuals such as the elderly, children, and those with compromised immune systems. Similarly, microcapsules containing polysaccharides with anti-tumor activities have been developed for targeted release within the human body, reducing gastrointestinal irritation in cancer patients and providing them with renewed hope for a better quality of life. 

The utilization of polysaccharides is closely tied to developments in science and technology. While modern instrument analytical techniques can effectively characterize polysaccharides, noteworthy progress has been attained in studying animal-derived polysaccharides. However, owing to the complex and unknown structure of polysaccharides, research is centered on the primary structural level. The lack of information on more advanced structures leads to the delayed development of pharmacological mechanisms and clinical behaviors and limits their applications and promotions. Furthermore, research has demonstrated that animal-derived polysaccharides have minimal utility in clinical treatment. Although animal polysaccharides are actively examined in laboratory research, industrial applications demand further expansion. 

## Figures and Tables

**Table 1:** Polysaccharides from various animals.

Sources	Type	Extraction Methods	Purification Methods	Crude Extraction Rate (%)	Molecular Weights (kDa)	References
Skin of *Rana dybowskii* Guenther	GAG	HW	Sepharose^TM^CL-6B	-	141	[31]
Sea cucumber intestines	Polysaccharide	HW	-	4.43	4.91 × 10^6^	[32]
*Aspongopus chinensis*	Polysaccharide	HW	-	5.067	-	[33]
Loach head	Polysaccharide	A	Sephadex G150	2.41	7.11 × 10^5^	[34]
Sturgeon cartilage	CS	E	-	5.7	111.48	[35]
Abalone gonad	Sulfated polysaccharide	PE	Source 30Q and Sephacryl-S 100,200	-	12.5	[36]
Codfish (*Gadus macrocephalus*) bones	CS	APE	DEAE Sepharose™Fast Flow	0.05	12.3	[37]
Sea cucumber viscera	Sulfated polysaccharide	PE	DEAE-52 Sephadex G-200	4.9	18.8	[38]
*Dosidicus gigas* cartilage	CS E	Actinase E enzymolysis	DEAE-Sephadex	15.3	696	[39]
*P. graeffei* *S. tremulus* *H. vagabunda* *I. badionotus* (sea cucumber body wall)	fCS	PE	DEAE-52	11.0 6.3 7.0 9.9	73 81 100 109	[40]
Body wall of sea cucumber *Stichopus herrmanni*	FG	PE	FPA98 (OH-)	7.5	6.37 × 10^4^	[41]
*Ruditapes philippinarum*	GAG	NPE	DEAE-650M	-	-	[42]
Razor clam	GAG	TE	DEAE-52	2.51	-	[43]
*Mactra veneriformis*	GAG	NP and TE	-	0.448	-	[44]
*Bullacta exarata*	GAG	N, P, and TE	-	0.265	-	[45]
*Patinopecten yessoensis* viscera	Polysaccharide	T and PSE	DEAE, Sepharose CL-6B	1	63	[46]
Tilapia head, fins, spine, and tail	CS	Savinase 16L and 2709 alkaline protease SE	-	1.02 0.81 0.38 0.93	1.94065 × 10^5^ 1.30967 × 10^5^ 1.07574 × 10^5^ 1.61347 × 10^5^	[47]
Muscle, viscera, and ovum of Chinese White Jade Snail	GAG	T and PSE	-	1.961 0.5731 0.7759	-	[48]
Buckskin	Polysaccharide	AP and PSE	-	1.231	-	[49]
*A. japonicus* and *H. mexicana* (sea cucumber body wall)	fCS	A—TCE	Q Sepharose Fast Flow	-	54.3 56.9	[50]
Sea urchin gonad	Polysaccharide	HW—E/HW—A—E	DEAE Fast Flow Sephadex G75, G200	11.1 6.2 9.4 9.0	4–600	[51]
Snail *Achatina fulica* mucus	GAG	AP—ACE	FPA98 (OH-)	10	3.343 × 10^5^	[52]
*Cipangopaludina chinensis*	Polysaccharide	Ultrasonic synchronous coupling papain enzymolysis	Q Sepharose Fast Flow Sephacryls-400	13.57	91.1	[53]
Vitreous body tilapia	HA	Ultrasonic—compound proteinase enzymolysis	HiTrap^TM^ DEAE-FF	11.44	100	[54]

GAG: glycosaminoglycan; CS: chondroitin sulfate; fCS: fucosylated chondroitin sulfate; FG: fucosylated glycosaminoglycan; HA: hyaluronic acid; HW: hot water; A: alkaline; E: enzymolysis; P: papain; AP: alkaline protease; NP: neutral protease; T: trypsin, SE: secondary enzymolysis; CE: combine enzymolysis; DEAE: diethylaminoethyl cellulose.

**Table 2:** Monosaccharides from various animals.

Sources	Type	Monosaccharide Composition	Proportion of Monosaccharides	References
Skin of *Rana dybowskii* Guenther	GAG	Man, GlcA, Glc, GalN, and Gal	0.41:2.67:0.16:1:0.25	[31]
Sea cucumber intestines	Polysaccharide	Man, Ara, Gal, Glc, and Fuc	22.3%, 19.31%, 11.78%, 3.22%, and 42.57%	[32]
Abalone gonad	Sulfated polysaccharide	Gal, Fuc, and Rha	46.9%, 32.7%, and 20.4%,	[36]
Sea cucumber viscera	Sulfated polysaccharide	Man, GlcN, GlcA, GalNAc, Glc, Gal, and Fuc	1.00:1.41:0.88:2.14:1.90:1.12:1.24	[38]
*P. graeffei* *S. tremulus* *H. vagabunda* *I. badionotus* (sea cucumber body wall)	fCS	GlcA, GalNAc, and Fuc	1.0:0.8:1.5 1.0:1.1:0.9 1.0:0.8:1.2 1.0:0.7:0.9	[40]
Body wall of sea cucumber *Stichopus herrmanni*	FG	GlcA, GalNAc, and Fuc	1:1.13:1.09	[41]
Sea urchin gonad	Polysaccharide	Man, GlcN, and Glc	5.18:1.00:4.13 7.78:1.91:1.00 5.06:1.00:8.01	[51]
Snail *Achatina fulica* mucus	GAG	GalNAc and IdoA	-	[52]

Man: mannose; GlcN: glucosamine; Glc: glucose; Rha: rhamnose; GlcA: glucuronic acid; GalN: galactosamine; Gal: galactose; Fuc: fucose; Ara: arabinose; GalNAc: N-acetylgalactosamine; and IdoA: iduronic acid.

**Table 3:** Critical data in animal polysaccharide bioactivity experiments.

Sources	Type	Experimental Model	In Vitro/In Vivo	Experimental Data	Biological Activity	References
Skin of *Rana dybowskii* Guenther	GAG	Mice	In vitro	50–400 μg/mL, T-lymphocyte proliferation ↑ 200–400 μg/mL, B-lymphocyte proliferation ↓	Immunological activity	[31]
*Aspongopus chinensis*	Polysaccharide	Mice breast cancer cells 4T1 Human breast cancer cells MDA-MB-453, MCF-7	In vitro	24 h, IC_50_: 10.260, 14.170, and 6.986 mg/mL 48 h, IC_50_: 5.719, 8.99, 6.302 mg/mL	Anti-tumor activity	[33]
Codfish (*Gadus macrocephalus*) bones	CS	-	In vitro	0.5 μg/mL, clotting times: 52 s and 25 s 50 μg/mL, clotting time: 46.3 s and 19.9 s	Anticoagulant activity	[37]
Sea cucumber viscera	Sulfated polysaccharide	Mice macrophage cells RAW264.7	In vitro	400 μg/mL, IL-1β,IL-6,TNF-α: 28.79, 289.36, and 673.56 pg/mL ↑	Immunological activity	[38]
*Dosidicus gigas* cartilage	CS E	Mice	In vivo	50–100 μg/mouse, 4T1 cancer cells metastasizing to the lung ↓	Anti-tumor activity	[39]
Tilapia head, fins, spine, and tail	CS	*Caenorhabditis elegans*	In vivo	0–1 mg/mL, lifespan of C. elegans increased from 12.33 to 18.50 h	Anti-aging activity	[47]
Sea urchin gonad	Polysaccharide	Macrophage cells RAW264.7	In vitro	6.25–800 μg/mL, IL-6, COX-2, TNF-α ↑	Immunological activity	[51]
Skin of gray triggerfish and smooth hound	sulfated polysaccharide	-	-	50 mg/mL, *Enterobacter* sp., *Salmonella enterica* ↓ 150 mg/mL, *Alternaria solani, Fusarium solani* ↓	Antibacterial activity	[72]
Loach ***(****Misgurnus anguillicaudatus)*	Polysaccharide	Mice	In vivo	100 and 200 mg/kg, serum insulin levels modest but significant elevation	Hypoglycemic activity	[77]
Sea cucumber	sulfated polysaccharides	Mice	In vivo	40 mg/kg, HDL-C levels, 67.7%, 36.7%, and 25.8% ↑ LDL-C levels, 13.2%, 32.9%, and 19.8% ↓	Hypolipidemic activity	[78]
Hybrid sturgeon cartilage	SCS	Mice	In vivo	800 μg/g, tumor biomarkers CEA decreased from 1237.22% to 612.48%, expression of CA19-9 decreased from 57.37% to 17.95%	Anti-tumor activity	[79]
*Apostichopus Japonius*	GAG	Mice	In vitro	0.5–10 μg/mL, Splenic lymphocytes proliferation ↑	Immunological activity	[80]

↑: facilitation, ↓: inhibition.

## Data Availability

Data are contained within the article.
